# MSTO1 is a cytoplasmic pro‐mitochondrial fusion protein, whose mutation induces myopathy and ataxia in humans

**DOI:** 10.15252/emmm.202317911

**Published:** 2023-07-11

**Authors:** Aniko Gal, Peter Balicza, David Weaver, Shamim Naghdi, Suresh K Joseph, Péter Várnai, Tibor Gyuris, Attila Horváth, Laszlo Nagy, Erin L Seifert, Maria Judit Molnar, György Hajnóczky

## Abstract

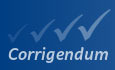

**Figure 1C and D, and Table EV1 are retracted along with their corresponding legend.**

**The associated claims regarding the presence of MSTO1 mutation c.22 G > A (p.Val8Met) in the investigated patients and the direct link between this mutation and patients' myopathy and ataxia phenotypes are retracted.**

**“MSTO P1” and “MSTO P2” are changed into “P1” and “P2,” respectively, throughout the manuscript.**

Journal Statement

Editors received a Correspondence article in April 2022, which was revised and published in July 2023 (Gerber *et al*, 2023). This Correspondence article raised concerns about an MSTO1 gene mutation that was identified and reported to be pathogenic in this manuscript, suggesting that the reported patient‐derived mutation is originated from a pseudogene MSTOP2 and not the protein‐coding gene MSTO1. In response, the editors contacted the authors of this manuscript, and Gal *et al* provided a brief response to the Correspondence article (Gai *et al*, 2023). Based on the peer‐reviewed results of the Correspondence, the author's response, the original manuscript, as well as further analysis conducted by the Journal, the editors decided to retract the above figures and associated claims.

PB, SJ, PV, AH, and GH agreed with this corrigendum. No response could be obtained from AG, DW, SN, TG, LN, ES, and MM.

All changes are in **“bold**.”

The “Title” on page 967 has been changed from “MSTO1 is a cytoplasmic pro‐mitochondrial fusion protein, whose mutation induces myopathy and ataxia in humans.” to “**MSTO1 is a cytoplasmic pro‐mitochondrial fusion protein.”**

The Abstract on page 967 has been changed from: “The protein MSTO1 has been localized to mitochondria and linked to mitochondrial morphology, but its specific role has remained unclear. We identified a c.22 G > A (p.Val8Met) mutation of MSTO1 in patients with minor physical abnormalities, myopathy, ataxia, and neurodevelopmental impairments. Lactate stress test and myopathological results suggest mitochondrial dysfunction. In patient fibroblasts, MSTO1 mRNA and protein abundance are decreased, mitochondria display fragmentation, aggregation, and decreased network continuity and fusion activity. These characteristics can be reversed by genetic rescue. Short‐term silencing of MSTO1 in HeLa cells reproduced the impairment of mitochondrial morphology and dynamics observed in the fibroblasts without damaging bioenergetics. At variance with a previous report, we find MSTO1 to be localized in the cytoplasmic area with limited colocalization with mitochondria. MSTO1 interacts with the fusion machinery as a soluble factor at the cytoplasm‐mitochondrial outer membrane interface. After plasma membrane permeabilization, MSTO1 is released from the cells. Thus, an MSTO1 loss‐of‐function mutation is associated with a human disorder showing mitochondrial involvement. MSTO1 likely has a physiologically relevant role in mitochondrial morphogenesis by supporting mitochondrial fusion.”

to: **“The protein MSTO1 has been localized to mitochondria and linked to mitochondrial morphology, but its specific role has remained unclear. Lactate stress test and myopathological results suggest mitochondrial dysfunction. In patient fibroblasts, MSTO1 mRNA and protein abundance are decreased and mitochondria display fragmentation, aggregation, and decreased network continuity and fusion activity. Short‐term silencing of MSTO1 in HeLa cells reproduced the impairment of mitochondrial morphology and dynamics observed in the fibroblasts without damaging bioenergetics. At variance with a previous report, we find MSTO1 to be localized in the cytoplasmic area with limited colocalization with mitochondria. MSTO1 interacts with the fusion machinery as a soluble factor at the cytoplasm‐mitochondrial outer membrane interface. After plasma membrane permeabilization, MSTO1 is released from the cells. MSTO1 likely has a physiologically relevant role in mitochondrial morphogenesis by supporting mitochondrial fusion.”**

The following text has been removed from the header of pages 968–984: “Linking of MSTO1 to myopathy and ataxia.”

The following text on page 968 has been removed: “In this study, we have identified an MSTO1 mutation in patients by whole‐exome sequencing. This mutation is segregated in the affected family and is absent in the other sequenced cases.”…“These symptoms and laboratory tests indicate a possible role for the newly found MSTO1 mutation in the background of mitochondrial disorders.”… “genetic rescue and”… “and its loss likely causes a multisystem disorder.”

The Fig 1. Legend on page 969 has been changed from “Figure 1. Clinical and genetic data of the patient.” to **“Figure 1. Clinical data of the patient.”**

The following text on pages 969 and 970 has been removed: “Finally, four missense mutations were validated with Sanger sequencing. Among them, only the c.22 G > A (p.Val8Met) substitution in MSTO1 gene is segregated in all affected family members and was present in heterozygous form (Table EV1 and Fig 1C). This mutation was found in urinary tract and colorectal tumors, as a somatic mutation (COSM3930426, COSM3930426) (http://cancer.sa nger.ac.uk); according to the Exome Aggregation Consortium (ExAC) database (http://exac.broadinstitute.org), the minor allele frequency is 0.003% (rs762798018), and it was absent in 1000 Genome (http://www.1000genomes.org), NHLBI Exome Sequencing Project (ESP; http://evs.gs.washington.edu/EVS/), ClinVar (http://www.ncbi.nlm.nih.gov/clinvar), dbGAP (http://www.ncbi.nlm.nih.gov/gap), and EGA (http://www.ebi.ac.uk/ega) databases. Connection with any clinical phenotype has not been described, yet. The mutated part of MSTO1 protein sequence is highly conserved in mammals (Fig 1D). This alteration was confirmed by cDNA sequencing from fibroblast as well (Fig 1C). Other alterations of MSTO1 gene were excluded by Sanger sequencing of the total coding sequence from genomic DNA and cDNA sequencing from patient derivate fibroblasts (MSTO P1, II/1, and MSTO P2, I/2). The copy number alteration was also excluded by real‐time PCR methodology.”…“G300E point mutation in the GTPase domain and D58 deletion of the GTPase domain caused 60–70% decrease in the fusion activity, whereas the present MSTO1 mutations induced an approximately 40% decrease in fusion activity. However, we have also studied an ADOA‐associated OPA1 mutation (c.984) that caused a lesser decrease in the fusion activity (30%) than the present MSTO1 mutations. Thus, the MSTO1‐associated fusion decrease is in the range of the OPA1‐associated ones.”

The following text on pages 971 and 973 has been removed: “MSTO1‐deficient.”

The Fig 4. Legend on page 974 has been changed from “Figure 4. MSTO1 overexpression in MSTO1 patient fibroblasts.” to **“Figure 4. MSTO1 overexpression in patient fibroblasts.”**

The following text on page 979 has been removed: “Sequencing of DNA isolated from the blood (whole‐exome and Sanger sequencing) assumed that Val8Met mutation in the MSTO1 gene is responsible for the clinical symptoms in the present family. The cDNA sequencing confirmed the heterozygous V8M, while in the genomic DNA, no exon loss or multiplication was found in this gene. Thus, the Hungarian patients seem to have one impaired and one normal MSTO1 allele, which results in a partial decrease in MSTO1 mRNA and protein levels. Based on the combination of several criteria, no other known pathogenic mutation is segregated in this family, but we cannot exclude that the partial loss of MSTO1 comes together with some other factors to cause the patients’ clinical picture.”

The following text on page 980 has been removed: “The cause–effect relationship between MSTO1 decrease and fusion inhibition was validated by genetic rescue studies in the patient fibroblasts (Fig 4).”…“Since a loss of function mutation in MSTO1 is associated with impaired mitochondrial dynamics and several symptoms characteristic to mitochondrial disease patients, MSTO1 might be a factor in diseases that have a mitochondrial component.”

The following text on page 983 has been removed from The Paper Explained: “This work reveals the first dominant mutation in MSTO1 associated with multisystemic disease in a Hungarian family.”…“Impact: Thanks to a multidisciplinary collaboration among clinicians, geneticists, and cell biologists, MSTO1 is found to be a new disease‐associated gene responsible for a rare mitochondrial multisystemic disorder.”
